# Association mapping and candidate gene identification for drought tolerance in sorghum

**DOI:** 10.3389/fpls.2025.1629615

**Published:** 2025-07-25

**Authors:** Huiting Min, Kang Wang, Tiantian Wang, Xinxiu Cheng, Ephrem Habyarimana, Yongfei Wang, Die Hu, Yi-Hong Wang, Lihua Wang

**Affiliations:** ^1^ College of Agriculture, Anhui Science and Technology University, Chuzhou, Anhui, China; ^2^ International Joint Research Center of Forage Bio-Breeding in Anhui Province, Chuzhou, China; ^3^ The International Crops Research Institute for the Semi-Arid Tropics (ICRISAT), Hyderabad, Telangana, India; ^4^ Department of Biology, University of Louisiana at Lafayette, Lafayette, LA, United States

**Keywords:** sorghum, mini core, GWAS, SNPs, drought tolerance, candidate genes

## Abstract

**Introduction:**

Water is essential for plant growth, and drought is one of the most predominant constraints on crop yield. Sorghum is a well-known drought-tolerant crop model, and sorghum landraces possess novel alleles for local adaptation.

**Methods:**

In this study, we evaluated a sorghum mini core panel of 239 landraces sampled globally for shoot and root growth under simulated drought conditions using 10% and 20% polyethylene glycol (PEG) in 2020 and 2024, and measured drought tolerance using the seedling tolerance coefficient (STC).

**Results and discussion:**

Phenotypic analysis showed that more accessions produced more roots than longer roots when exposed to 10% PEG; however, at 20% PEG, more accessions produced longer roots than more roots, reflecting the adaptability of some accessions to drought stress. However, PEG reduced shoot growth in all accessions in both years. A genome-wide association study (GWAS) on 32 growth and 19 STC traits identified 22 loci, 19 of which were mapped to the STC traits, and 17 of these 19 were associated with STC of shoot weight. Eleven of the 22 loci were collocated with 23 previously identified mapped drought-related quantitative trait loci (QTLs); 15 of these 23 QTLs were mapped to green leaf area, total number of green leaves, or chlorophyll content. We also found 19 candidate genes for 12 of the 22 loci. Five of those genes showed either preferential or specific expression in the roots according to GeneAtlas v2. One candidate gene from a locus colocated with a previously mapped chlorophyll fluorescence QTL has been shown to increase chlorophyll fluorescence in maize in another study. The results of this study lay the foundation for further characterizing the sorghum mini core panel for novel drought-tolerant genes.

## Introduction

Water is critical for plant growth and development. As with all crop plants, the growth of sorghum [*Sorghum bicolor* (L.) Moench] relies on an adequate water supply in the form of rainfall or irrigation well distributed throughout the growing season (Assefa et al., 2010; Eck and Musick, 1979). For example, a medium-to-late sorghum variety maturing between 110 and 130 days would require approximately 450 to 650 mm of water during the growing season, and for this reason, the average yield of dryland sorghum is approximately half that of irrigated sorghum (Assefa et al., 2010). Not surprisingly, the total water supply (available soil water at seedling emergence plus in-season precipitation) is significantly correlated with sorghum grain yield (*r* ^2^ = 0.834), and for every centimeter increase of available soil water at seedling emergence and in-season precipitation, sorghum grain yield increases by 221 and 164 kg ha^−1^, respectively (Stone and Schlegel, 2006). This indicates that soil water at seedling emergence is slightly more important for grain yield, probably because the early stages of plant growth (germination, emergence, and seedling establishment) are potentially the most vulnerable to drought stress (Abreha et al., 2022).

Despite yield reduction by drought, sorghum is considered more drought-resistant than many other crop plants (Hadebe et al., 2017) and shows a wide range of morphological, physiological, and biochemical adaptations in response to drought stress (Liu et al., 2024). When exposed to drought, older sorghum leaves are selectively killed, while the younger leaves remain physiologically functional as a result of osmotic adjustment in the younger leaves (Blum, 2005). Sorghum plants can have higher water use efficiency because they can reduce evapotranspiration more efficiently (Tolk and Howell, 2003). This is most likely because drought-tolerant sorghums tend to produce more epicuticular wax on their leaf surface compared to sensitive ones during drought stress (Sanjari et al., 2021). Further support comes from overexpressing a sorghum *WINL1*, which simultaneously increases total wax/cutin content and drought tolerance in *Arabidopsis* (Bao et al., 2017). Another reason may be that during drought, drought-tolerant sorghum plants show more leaf rolling than the susceptible lines, reducing the effective evapotranspiration area of the uppermost leaves by approximately 75% (Matthews et al., 1990). In addition to this leaf feature, sorghum plants tend to penetrate deeper into the subsoil (40–135 cm) (Schittenhelm and Schroetter, 2014; Singh and Singh, 1995), and more roots are produced during drought (de Oliveira et al., 2022; Queiroz et al., 2019). A combination of these two factors may account for up to 90% of the total water used by sorghum (Rachidi et al., 1993). Therefore, this root feature (more and deeper roots) has been found to be a major contributor to drought tolerance in sorghum (Wright and Smith, 1983). At the physiological level, drought induces the large central vacuole to form small vesicles when the leaf water potential is at −37 bars; this maintains tonoplast integrity and allows sorghum plants to withstand drought (Giles et al., 1976). It is not surprising that drought elicits extensive genetic (Abreha et al., 2022) and proteomic responses (Li et al., 2020) in sorghum.

Because of its importance, drought tolerance has been extensively mapped in sorghum. By searching drought tolerance-related traits in the Sorghum quantitative trait locus (QTL) Atlas (Mace et al., 2019), 817 loci were identified from 19 studies published before 2019. More recently, Tsehaye et al. (2024) mapped 32 drought-related quantitative trait nucleotides (QTNs) using an association mapping panel of 216 diverse accessions and 17,637 Single Nucleotide Polymorphism (SNP) markers, four of which colocated with previously mapped drought-related QTLs. Faye et al. (2022) mapped 16 pleiotropic associations for drought responses across water stress environments using an association mapping panel of 590 predominantly West African sorghum landraces and 130,709 SNPs. Crop landraces represent local adaptations of domesticated species and contribute novel alleles for adaptation to stressful environments (Dwivedi et al., 2016). Although larger panels (Faye et al., 2022; Tao et al., 2020, 2021) have been used in a sorghum genome-wide association study (GWAS), we found that the sorghum mini core panel is effective in QTL mapping. In a previous study, we mapped and cloned a pleiotropic QTL gene for plant height, days to 50% flowering, biomass, juice yield, and juice sugar content (Upadhyaya et al., 2022) using the sorghum mini core collection of 242 global landraces (Upadhyaya et al., 2009). The mini core panel has since been used to map sorghum panicle architecture (Wang et al., 2021), sorghum plant color (Wang et al., 2024), callus induction and regeneration from mature sorghum seeds (Xu et al., 2025), and additional developmental and reproductive traits (Upadhyaya et al., 2024).

In this study, the objective was to map drought tolerance loci that are pleiotropic for more than one trait or stable across environments. Drought stress was imposed by polyethylene glycol (PEG), which is commonly used to simulate drought in sorghum (Abdel-Ghany et al., 2020; Dugas et al., 2011; Jafar et al., 2004; Pavli et al., 2013; Queiroz et al., 2019), on the sorghum mini core panel. To carry out the mapping, we evaluated seedling shoot/root length, shoot/root fresh/dry weight, germination rate with and without osmotic stress, and drought indices, which were calculated as the ratio of growth under stressed and control conditions in 2020 and 2024 and performed a GWAS on the traits as previously described (Li et al., 2018) using 6,094,317 SNP markers (Upadhyaya et al., 2022; Wang et al., 2021; Xu et al., 2025). We identified 17 QTLs for shoot fresh and dry weight and drought index, along with five QTLs for other traits. A suite of candidate genes landed on by or closest to linked SNPs was also identified.

## Materials and methods

### Plant materials and osmotic stress assay

A mini core panel of 239 accessions (Upadhyaya et al., 2009) was used for this study. Uniform, full, and healthy seeds, free from mechanical damage or pest or disease infection, were used. The selected seeds were surface-sterilized with a 0.1% mercuric chloride (HgCl_2_) solution for 15 minutes and then rinsed thoroughly three times with sterile distilled water to remove any residual HgCl_2_. The sterilized seeds were treated with 10% and 20% polyethylene glycol (PEG-6000) solutions in 2020 (20_10 and 20_20, respectively) and with 10% PEG in 2024 (24_10) to simulate drought stress, with distilled water as the control. The 20% PEG treatment was not repeated in 2024 because it interfered with germination and subsequent seedling growth. Each treatment consisted of 30 seeds for each of the three replicates in each accession. Both the control and treatment seeds germinated on two pieces of special blotting paper (12 × 12 cm) in a germination box and were incubated in a plant growth chamber at a constant temperature of 28°C with a 16-hour light/8-hour dark photoperiod for 10 days. On the 10th day, 10 uniformly growing seedlings were selected from each treatment. Shoot length (SL) and root length (RL) were measured using a ruler to the nearest millimeter. Fresh weights of shoots and roots (SFW and RFW, respectively) were determined using an electronic balance with a precision of 0.0001g. The shoots and roots were then dried at 75°C for 24 hours and cooled to room temperature, and their dry weights (SDW and RDW, respectively) were measured using the same balance. The germination rate (GR) was recorded for 2024.

The drought tolerance was assessed using the seedling tolerance coefficient (STC) (Qiu et al., 2007; Yu et al., 2021) and was calculated as follows:


STC=(Measured value under treatment/Measured value under control)

The STC calculated for each trait was denoted as RL_STC_ for root length, while root length for the 10% or 20% PEG treatment conducted in 2020 was denoted as RLPEG20_10 and RLPEG20_20, respectively; the control was denoted as RL20. A list of all traits is provided in **Supplementary Table S1**.

### Association mapping

GWAS was conducted as described (Li et al., 2018; Upadhyaya et al., 2022, 2024; Wang et al., 2021, 2024; Xu et al., 2025). In short, GWAS for the 51 traits (listed together with all Manhattan plots in **Supplementary Figure S1** and **Supplementary Table S1**) was performed using 6,094,317 SNPs. A kinship matrix (K) was generated with EMMAX (Kang et al., 2010), and a Q matrix was calculated using STRUCTURE 2.3.4 (Pritchard et al., 2000). Both matrices were used to perform GWAS in an Mixed Linear Model (MLM) model (Yu et al., 2006). The modified Bonferroni correction was used to determine association significance thresholds. At a nominal level of α = 0.05, the threshold *p*-value was 8.2 × 10^−9^, or a −log_10_(*p*) value of 8.08. As in previous studies (Upadhyaya et al., 2022; 2024), we also included markers with *p*-values below 10^−4^ (Famoso et al., 2011; Zhao et al., 2011) to account for associations of multiple markers at a locus across more than two traits to declare an association.

### QTL colocalization and identification of candidate genes

As described by Upadhyaya et al. (2024), to identify colocalizing QTLs mapped in this study based on physical location, previously mapped QTLs downloaded from the Sorghum QTL Atlas (Mace et al., 2019) and those by Faye et al. (2022) were used. The location of candidate genes was identified with the BTx623 reference sequence (whose complete genome is now available; Wei et al., 2024), *S. bicolor* v3.1.1 (McCormick et al., 2018) at Phytozome 13 (Goodstein et al., 2012). Genes, including linked SNP markers with *p*-values below 10^−4^, were considered candidate genes based on previous studies that showed that linked markers can land on the causal genes (Upadhyaya et al., 2022; Wang et al., 2016; Zhang et al., 2023).

### Statistical analysis

Pearson’s correlation coefficient (*r*) was calculated using Excel’s PEARSON function. Its significance was tested using a table of critical values. The assumptions of analysis of variance (ANOVA), i.e., data normality and variance homogeneity, were confirmed using the Kolmogorov–Smirnov test (see **Supplementary Table S2**). ANOVA was performed using the SPSS V29.0 statistical software with a general linear model. The variance components generated from the ANOVA were used to calculate the broad-sense heritability (*H* ^2^) for the RL_STC_, RDW_STC_, RFW_STC_, SL_STC_, SDW_STC_, and SFW_STC_ using the following formula:


$$H^{2}=Vg/\left(Vg+Vge+Ve\right)$$

where *Vg*, *Vge*, and *Ve* are genetic variance, the genotype × environment interaction variance, and environmental variance, respectively (Smith et al., 1998; Upadhyaya et al., 2024).

## Results

### Phenotypic analysis

We analyzed phenotypic variation among replicates for each accession. We found that if ranked by minimal dispersion using standard deviation (SD) for SLPEG20_10, three of the top four accessions (IS12302, IS20697, and IS2382) were all caudatum, and one (IS30466) was caudatum-bicolor (**Supplementary Table S3**). Pearson’s correlation between the STC traits of SFW_STC_20_20 and SDW_STC_20_20 was highest (*r* = 0.95; *r* = 0.77) between SFW_STC_20_10 and SDW_STC_20_10 and was *r* = 0.82 between SFW_STC_24_10 and SDW_STC_24_10; all significant at *p* < 0.001. We also observed a similar trend between RFW_STC_ and RDW_STC_ (**Supplementary Table S4**). This was followed by SL_STC_ and SFW_STC_, and SL_STC_ and SDW_STC_ (both 0.84, significant at *p* < 0.001). We also found that SDW_STC_20_20 was highly and significantly correlated with RDW_STC_20_20, RFW_STC_20_20, and SL_STC_20_20 with *r* of 0.8, 0.77, and 0.84, respectively (**Supplementary Table S4**). Interestingly, RDWPEG20_10 was more highly correlated with SL20, RL20, SFW20, RFW20, SDW20, and RDW20 than with RDWPEG20_20 (**Supplementary Table S4**).

We found that 34%–55% of the mini core panel produced more root biomass and longer roots during osmotic stress (**Table 1**). At 10% PEG, more accessions produced more roots (47%–55%) than longer roots (19%), and this trend was also observed in the 2024 data; however, at 20% PEG, more accessions produced longer roots (34%) than more roots (13%–20%). On average, in 2020, the 10% PEG treatment reduced RL by 20%, and the 20% PEG treatment reduced RL by 72%. In 2024, when only 10% PEG was used, RL was reduced by 34% due to the treatment. When ranked by RL_STC_, none of the bottom 70 accessions produced longer roots in 2020 when treatment was increased from 10% to 20% PEG (**Supplementary Table S5**). Furthermore, no accessions invested in shoot growth at 20% PEG, although a few random accessions (2%–7%) did show increased shoot weight at 10% PEG in both years (**Table 1**). This clearly demonstrates that osmotic stress greatly reduces shoot growth. Interestingly, all three root STC traits, RL_STC_, RDW_STC_, and RFW_STC_, had slightly lower broad-sense heritability than the shoot traits (**Table 2**). This was most likely due to the non-genetic root response to osmotic stress, as the root was in direct contact with the stressor.

**Table 1:** Number of mini core accessions with increased phenotypic values by PEG treatments*.

Trait		Accessions (percentage)
RL_STC_	RL_STC_20-10	34 (19)
	RL_STC_20-20	60 (34)
	RL_STC_24_10	17 (10)
RDW_STC_	RDW_STC_20-10	98 (55)
	RDW_STC_20-20	23 (13)
	RDW_STC_24_10	30 (17)
RFW_STC_	RFW_STC_20-10	84 (47)
	RFW_STC_20-20	35 (20)
	RFW_STC_24_10	24 (13)
SL_STC_	SL_STC_20-10	2 (1)
	SL_STC_20-20	0 (0)
	SL_STC_24_10	9 (5)
SDW_STC_	SDW_STC_20-10	12 (7)
	SDW_STC_20-20	0 (0)
	SDW_STC_24_10	8 (4)
SFW_STC_	SFW_STC_20-10	4 (2)
	SFW_STC_20-20	0 (0)
	SFW_STC_24_10	10 (6)

*10% (20_10) and 20% PEG (20_20) in 2020 and 10% in 2024. A total of 179 accessions with missing data points of no more than one were included in the accession count and percentage calculation. Values in parentheses are percentages.

STC, seedling tolerance coefficient; SL/RL, shoot/root length; SFW/RFW, shoot/root fresh weight; SDW/RDW, shoot/root dry weight; PEG, polyethylene glycol.

**Table 2:** Broad-sense heritability (*H* ^2^) of the six seedling tolerance coefficient traits.

Trait		*H* ^2^	Average
RDW_STC_	RDW_STC_20-10	0.690558	0.709377
	RDW_STC_20-20	0.748958	
	RDW_STC_24_10	0.688615	
RFW_STC_	RFWSTC20-10	0.501197	0.642634
	RFWSTC20-20	0.737857	
	RFWSTC24_10	0.688847	
RL_STC_	RL_STC_20-10	0.754941	0.702646
	RL_STC_20-20	0.611755	
	RL_STC_24_10	0.741242	
SDW_STC_	SDW_STC_20-10	0.85714	0.792949
	SDW_STC_20-20	0.805177	
	SDW_STC_24_10	0.716529	
SFW_STC_	SFW_STC_20-10	0.907195	0.813592
	SFW_STC_20-20	0.765715	
	SFW_STC_24_10	0.767865	
SL_STC_	SL_STC_20-10	0.739947	0.780708
	SL_STC_20-20	0.746749	
	SL_STC_24_10	0.855427	

Based on RL_STC_ and RDW_STC_, we identified one drought-tolerant (IS 30533) and one sensitive (IS 32439) accession. On average, the IS 32439 root length and dry weight were reduced by the PEG treatment by 64% and 71%, respectively, while in IS 30533, these were increased by 20% and 19%, respectively. Still, for IS 30533, shoot length and dry weight were decreased by 40% and 37%, respectively, by the treatment, while they were 65% and 60%, respectively, for IS 32439 (**Supplementary Table S5**).

### Association mapping

We applied the same criterion used in previous studies (Upadhyaya et al., 2022, 2024; Wang et al., 2021) for trait mapping in the sorghum mini core panel, which defines a significant association as multiple SNPs linked to a trait within the same locus with a *p*-value < 10^−4^. In addition to identifying trait-specific loci, this study also focused on pleiotropic loci—those associated with more than one trait. With this criterion, we identified 22 loci linked to 12 drought-related traits (**Table 3**; all 51 Manhattan plots are provided in **Supplementary Figure S1**). SDW_STC_ and SFW_STC_ had the highest correlation coefficient (**Supplementary Table S4**). Here, we report that 17 of the 22 mapped loci were pleiotropic for the two traits and that 19 of the 22 loci were mapped to the STC traits (**Table 3**). Seven traits (RFW_STC_20_20, SL_STC_20_20, SDW_STC_20_20, SFW_STC_20_20, SFWPEG20_20, SDWPEG20_20, and SLPEG20_20) were all mapped to the 4–1 locus. Loci 2–1 and 4-2 (RFW_STC_20_20, SL_STC_20_20, SDW_STC_20_20, and SFW_STC_20_20) and 6–1 and 6-2 (SDW_STC_20_20, SFW_STC_20_20, SDWPEG20_20, and SFWPEG20_20) were all pleiotropic for four traits, while all other loci were pleiotropic for two traits (**Table 3**).

**Table 3:** Drought tolerance-related loci mapped in the sorghum mini core panel and their colocation with previously mapped drought-related QTLs.

QTLs mapped in this study				Previously mapped QTLs			
ID	Top SNPs	Trait/−log(*p*) value		Location	Trait	Name	Reference
1-1		SDW_STC_20_20	SFW_STC_20_20	1:7709494–14555494 1:7780218–8980613 1:7895113–9046512 1:8086604–9156345 1:8180004–8187693	Total number of green leaves Plant height Biomass Fresh biomass Maturity	QTNGL1.2 QHGHT1.58 QBMAS1.9 QFBMS1.51 KN1	Rama Reddy et al., 2014; Spindel et al., 2018; Faye et al., 2022
	8175770	6.092572476	5.901292726				
	8175846	6.069833035	6.328635272				
	8181926	6.734819235	6.658063039				
1-2		SDW_STC_20_20	SFW_STC_20_20				
	25423417	6.696211597	5.046218177				
	25423421	6.896639032	5.274721862				
	25423654	6.351679731	6.124947452				
1-3		SFWPEG20_20	SLPEG20_20	1:60998392–61246047	Biomass at 65% moisture	QBM651.10	Spindel et al., 2018
	61062232	6.244060667	7.240034659				
	61067704	6.244060667	7.240034659				
	61067917	6.970580775	7.951858174				
2-1		RFW_STC_20_20	SL_STC_20_20	2:47479285–50768059 2:48465917–51754692 2:47540401–57021411	Plant height Fresh biomass Green leaf area	QHGHT2.22 QFBMS2.12 QGLFA2.2	Spindel et al., 2018; Rama Reddy et al., 2014
	49420951	4.487674263	3.424231104				
	49421672	4.179738517	3.309300367				
	49422838	6.213284552	4.904262718				
		SDW_STC_20_20	SFW_STC_20_20				
	49420951	6.415666917	7.017774935				
	49421672	6.33486414	6.889629854				
	49422838	7.220320153	7.486824187				
3-1		SDW_STC_20_20	SFW_STC_20_20				
	20274965	7.283425462	5.58199737				
	20274987	6.01480676	4.077951971				
	20274991	6.494032608	4.525464497				
	20275019	7.098582705	5.276704707				
3-2		SDW_STC_20_20	SFW_STC_20_20	3:51390526–55538940 3:51629032–55604826 3:53876527–55222843 3:53884338–55533369 3:54220793–55419201	Green leaf area Total number of green leaves Total number of green leaves Green leaf area Green leaf area	QGLFA3.3 QTNGL3.3 QTNGL3.4 QGLFA3.5 QGLFA3.4	Rama Reddy et al., 2014
	54673890	5.864308749	5.34388905				
	54673977	5.864308749	5.34388905				
	54674484	6.282688315	5.576211248				
4-1		RFW_STC_20_20	SL_STC_20_20	4:71001–1548102	Green leaf area	QGLFA4.1	Srinivas et al., 2009
	335179	7.178732257	5.829212644				
	340675	7.223628346	5.582475776				
	340847	7.223628346	5.582475776				
		SDW_STC_20_20	SFW_STC_20_20				
	335179	8.549496331	7.423224934				
	340675	7.198161217	6.672253247				
	340847	7.198161217	6.672253247				
		SFWPEG20_20	SDWPEG20_20				
	335179	6.634126667	7.679019892				
	340675	6.781339929	7.46226058				
	340847	6.781339929	7.46226058				
		SLPEG20_20					
	335179	6.284284665					
	340675	7.312943179					
	340847	7.312943179					
4-2		RFW_STC_20_20	SL_STC_20_20				
	7340159	6.005381451	5.955787394				
	7340188	6.146902096	5.711189696				
		SDW_STC_20_20	SFW_STC_20_20				
	7340159	6.043780556	5.883865159				
	7340188	6.201848721	5.807850664				
5-1		SLPEG20_20	SLPEG20_10				
	14749135	7.20588014	4.310967245				
	14764206	7.031012426	4.726055663				
	14790465	8.295266962	6.3811091				
	14817605	7.741075943	5.659760658				
	14838313	7.22546815	4.465115849				
	14851504	8.017381333	4.50832729				
5-2		RFW20_20	RFWPEG20_10				
	20946132	9.032320539	7.242243694				
	20946153	8.476547344	7.418053564				
	20946253	7.053352114	4.882369306				
5-3		SDW_STC_20_20	SFW_STC_20_20				
	65929984	6.29492177	5.650168324				
	65930108	6.360960499	5.719394472				
6-1		SDW_STC_20_20	SFW_STC_20_20	6:1671780–1714417	Chlorophyll fluorescence	QCHLF6.8	Fiedler et al., 2014
	1683157	7.763763571	8.047866994				
	1683159	6.743550697	7.170180763				
	1683181	6.366959595	6.854304226				
		SDWPEG20_20	SFWPEG20_20				
	1683157	7.173383002	7.300309843				
	1683159	6.10531889	6.323799102				
	1683181	6.277580714	6.560284309				
6-2		SDW_STC_20_20	SFW_STC_20_20	6:46558064–50652065	Green leaf area	QGLFA6.1	Srinivas et al., 2009
	46599990	6.778232968	7.258141677				
	46602135	6.799929522	7.245531549				
	46613461	6.799929522	7.245531549				
	46613645	7.177554732	7.880833313				
		SDWPEG20_20	SFWPEG20_20				
	46599990	6.256926186	6.636795736				
	46602135	6.310414974	6.652851601				
	46613461	6.310414974	6.652851601				
	46613645	6.613541225	7.212360472				
7-1		GRCK2024	GR_STC_24_10				
	25776380	6.358062437	4.281133202				
	25777587	7.526355067	5.094466411				
	25777916	8.225453962	5.262387727				
7-2		SDW_STC_20_10	SFW_STC_20_10	7:39543566–48379318	Chlorophyll content	QCHLC7.4	Fiedler et al., 2014
	45098852	7.41519769	7.515380775				
	45098853	6.190476311	6.267296943				
8-1		SDW_STC_20_20	SFW_STC_20_20				
	4718439	10.14452745	8.496081295				
	4719344	6.388117926	5.886092378				
	4726212	7.220582719	5.798288488				
9-1		RFW20	SFWPEG20_10				
	734720	8.401017801	6.275968976				
	734771	7.915613864	4.374989053				
9-2		SFW_STC_24_10	RFW_STC_24_10				
	9798734	6.7147509	5.255642033				
	9799294	7.039341044	5.70565594				
	9800202	6.582200035	5.26023082				
9-3		RFW20_20	SFWPEG20_10	9:55713786–57908267 9:55813801–57991519 9:56104114–56610219	Green leaf area Total number of green leaves Chlorophyll fluorescence	QGLFA9.6 QTNGL9.1 QCHLF9.13	Sabadin et al., 2012; Rama Reddy et al., 2014; Fiedler et al., 2014
	56373379	8.025536867	4.280428888				
	56374198	6.378388946	4.424347292				
10-1		SDW_STC_20_10	SFW_STC_20_10				
	27918593	5.81835559	7.428463949				
	27918661	6.036030639	7.212369448				
	27918667	6.163314405	6.67572531				
10-2		SDW_STC_20_20	SFW_STC_20_20	10:41745111–52246823	Green leaf area	QGLFA10.2	Haussmann et al., 2002
	44310626	5.48809613	6.205218693				
	44329730	5.897243312	6.965896225				
	44351912	7.029334136	7.536690866				
	44366553	6.232655998	7.053328408				
	44427299	6.422957194	7.224057245				
10-3		SDW_STC_20_20	SFW_STC_20_20	10:45658506–51995783	Green leaf area	QGLFA10.3	Haussmann et al., 2002
	46853453	6.405686191	6.018533018				
	46853558	7.520008804	7.200200734				

See **Table 1** notes for other abbreviations.

1–1 etc.: the first digit indicates sorghum chromosome number, and the second the locus order number. QTL location before 2019 was from the Sorghum QTL Atlas (Mace et al., 2019).

GR, germination rate; CK, control.

### Colocation with previously mapped drought-related QTLs

Eleven (1-1, 1-3, 2-1, 3-3, 4-1, 6-1, 6-2, 7-2, 9-3, 10-2, and 10-3) of the 22 loci were colocated with 23 previously mapped QTLs (**Table 3**). Among the 23 QTLs, 15 were associated with drought-related leaf features: nine (QGLFA2.2, QGLFA3.3, QGLFA3.5, QGLFA3.4, QGLFA4.1, QGLFA6.1, QGLFA9.6, QGLFA10.2, and QGLFA10.3) were associated with green leaf area, four (QTNGL1.2, QTNGL3.3, QTNGL3.4, and QTNGL9.1) were associated with the total number of green leaves, and two (QCHLF6.8 and QCHLF9.13) were associated with chlorophyll content (**Table 3**). Among the 11 loci, 1–1 and 3–2 were each colocated with five QTLs, while 2–1 and 9–3 were each colocated with three QTLs; the rest were colocated with one QTL (**Table 3**).

### Candidate genes identified by linked SNPs

Candidate genes were identified because they had linked SNP markers that landed in coding, or 5′/3′ regions, or were closest to the linked SNPs. Using these criteria, we found 19 candidate genesmacross 12 of the 22 loci (**Table 4**). Five candidate genes –a transporter (Sobic.001G323600) in locus 1-3, a UDP-glucosyl transferase (Sobic.004G087300) in locus 4-2, and three aldo/keto reductases (Sobic.006G096000, Sobic.006G096100, and Sobic.006G096200) in locus 6-2 –showed consistently high expression in the roots based on data available from GeneAtlas v2 FPKM (McCormick et al., 2018). In addition, a nucleoporin gene (Sobic.006G011700) displayed shoot-specific expression (**Supplementary Table S6**). Haplotypes based on the three SNPs (46613461, 46613645, and 46615325) located in the promoter and coding regions of Sobic.006G096100 in locus 6-2 showed that IS 30533 had TTC while IS 34239 had CCT at these positions (**Supplementary Table S7**).

**Table 4:** Candidate genes identified in this study.

Locus	Top SNPs	Trait	Candidate gene
1-1	8175770 8175846 8181926	SDW_STC_20_20 SFW_STC_20_20	Sobic.001G106400 E3 ubiquitin-protein ligase FANCL Sobic.001G106200 HOMEOBOX PROTEIN KNOTTED-1-LIKE 1/KN1
1-3	61062232 61067704 61067917	SFWPEG20_20 SLPEG20_20	Sobic.001G323600 Polyol transporter Sobic.001G323500 DUF789
2-1	49420951 49421672 49422838	RFW_STC_20_20 SL_STC_20_20 SDW_STC_20_20 SFW_STC_20_20	Sobic.002G159900 chloroplastic phosphoenolpyruvate/phosphate translocator 1
4-1	335179 340675 340847	RFW_STC_20_20 SL_STC_20_20 SDW_STC_20_20 SFW_STC_20_20 SFWPEG20_20 SDWPEG20_20 SLPEG20_20	Sobic.004G004100 Pentatricopeptide (PPR) repeat-containing protein-like Sobic.004G004200 Regucalcin gene promoter region-related protein Sobic.004G003700 Myb_DNA-bind_4
4-2	7340159 7340188	RFW_STC_20_20 SL_STC_20_20 SDW_STC_20_20 SFW_STC_20_20	Sobic.004G087300 UDP-glucosyl transferase 73C
5-1	14749135 14764206 14790465 14817605 14838313 14851504	SLPEG20_20 SLPEG20_10	Sobic.005G094400
5-2	20946132 20946153 20946253	RFW20 RFWPEG20_10	Sobic.005G108300 jasmonic acid-amino synthetase (JAR1)
6-1	1683157 1683159 1683181	SDW_STC_20_20 SFW_STC_20_20 SDWPEG20_20 SFWPEG20_20	Sobic.006G011700 NUCLEOPORIN-RELATED
6-2	46599990 46602135 46613461 46613645	SDW_STC_20_20 SFW_STC_20_20 SDWPEG20_20 SFWPEG20_20	Sobic.006G096000 ALDO/KETO REDUCTASE Sobic.006G096100 ALDO/KETO REDUCTASE Sobic.006G096200 ALDO/KETO REDUCTASE
8-1	4718439 4719344 4726212	SDW_STC_20_20 SFW_STC_20_20	Sobic.008G047900 HSP20-like chaperone Sobic.008G048000 Auxin responsive protein
9-2	9798734 9799294 9800202	SFW_STC_24_10 RFW_STC_24_10	Sobic.009G075400 PROTEIN RALF-LIKE 4 Sobic.009G075300 DUF1677
10-1	27918593 27918661 27918667	SDW_STC_20_10 SFW_STC_20_10	Sobic.010G140600 BOLA-LIKE PROTEIN-RELATED

## Discussion

In this study, we evaluated a sorghum mini core panel for shoot and root growth under simulated drought conditions imposed by 10%/and 20% PEG. The results showed that certain accessions exhibited enhanced root growth—through either increased root number or elongation—under osmotic stress. A greater number of accessions produced more roots rather than longer roots when exposed to 10% PEG. However, at 20% PEG, more accessions exhibited longer roots than increased root number, reflecting the adaptability of some accessions to drought stress. These findings are consistent with those of previous studies (Bibi et al., 2010; de Oliveira et al., 2022; Schittenhelm and Schroetter, 2014; Singh and Singh, 1995). Based on the response of root length and dry weight (RL_STC_ and RDW_STC_), IS 30533 was identified as the most drought-tolerant, while IS 32439 was the most sensitive accession. These accessions may be of particular interest for sorghum breeding programs targeting improved drought tolerance.

Drought stress significantly and negatively impacts sorghum growth (Abreha et al., 2022), especially shoot growth (Jafar et al., 2004), which was most significantly reduced by PEG treatments as measured by shoot fresh and dry weight STC (SDW_STC_ and SFW_STC_) (**Table 1**). This explains why among the 22 loci identified, 19 were mapped to the STC traits, which reflect the impact on growth, and 17 were mapped to SDW_STC_ and SFW_STC_. Half of the mapped loci are also colocated with 23 previously mapped drought-related QTLs; 15 of these 23 QTLs were mapped to green leaf area, total number of green leaves, or chlorophyll content (**Table 3**). We also found 19 candidate genes for 12 of the 22 loci. Five of those genes show either preferential or specific expression in the roots (**Supplementary Table S6**). The relevance of some of these candidate genes to drought tolerance is explained in the following sections.

When exposed to drought stress, the immediate response must be to protect the cell. One candidate gene identified in locus 8–1 encodes Hsp20, which has been found to be induced by drought stress in sorghum (Abdel-Ghany et al., 2020; Zhang et al., 2024). As a small heat shock protein, Hsp20, may form a complex with a variety of non-native proteins to form a first line of defense against protein aggregation during stress (Haslbeck and Vierling, 2015). Drought also induces transporters for various solutes (Dong et al., 2014), and we found one polyol transporter in locus 1-3 and a chloroplastic phosphoenolpyruvate/phosphate translocator (PPT) in locus 2-1. a polyol transporter is an H^+^-dependent plasma membrane carrier that transports mannitol and sorbitol, which protect cells against osmotic stress (Shen et al., 1999) and are induced in grapevines by drought (Conde et al., 2015). PPT imports phosphoenolpyruvate (PEP) to the plastid from the cytosol. A loss-of-function mutant of PPT1 in *Arabidopsis* results in stunted roots (Staehr et al., 2014), potentially compromising the ability of roots to cope with drought. Regarding transporters, we identified in locus 6 -1 a nucleoporin that is the main transport channel between the cytoplasm and the nucleoplasm, and a maize nucleoporin, *ZmNUP58*, has been shown to play an important role in the stress response of maize. *ZmNUP58* overexpression in maize significantly promotes both chlorophyll content and activities of antioxidant enzymes under drought conditions (Liu et al., 2022). Coincidentally, a chlorophyll fluorescence QTL (QCHLF6.8) (Fiedler et al., 2014) is also colocated in this locus (**Table 3**), demonstrating the effectiveness of drought QTL mapping using the mini core panel in this study. In sorghum, the stay-green trait contributes to the adaptation to post-flowering drought conditions (Abreha et al., 2022). Since drought reduces sorghum leaf chlorophyll content (Kapanigowda et al., 2013), increased chlorophyll content during drought is a sign of drought tolerance (Kassahun et al., 2010). For this reason, we found at least five QTL clusters from six studies in which stay-green loci overlap with chlorophyll content loci: one each on chromosomes 2 and 10, and three on chromosome 3 (**Supplementary Table S8**).

Sorghum also exhibits physiological and biochemical resistance to drought by scavenging reactive oxygen species (ROS) and changing the activity of its antioxidant enzymes (Liu et al., 2024). For ROS scavenging, a BolA protein identified in locus 10 -1 may play a negative role in ROS scavenging, as a mutation in *Arabidopsis BolA* causes the plant to produce longer roots and to scavenge ROS, implying an increased capacity to extract deeper soil water (Qin et al., 2015). Another example of a ROS scavenger (Yu et al., 2020) is the three aldo-keto reductases (AKR) in locus 6-2, which are mostly expressed in the roots (**Supplementary Table S6**). In tomatoes, the majority of *AKR* genes are induced by drought treatments, and silencing *AKR* expression reduces drought tolerance due to low proline content and high malondialdehyde content, indicating *AKR*s’ positive role in regulating drought tolerance in tomatoes (Guan et al., 2023). So is the E3 ubiquitin-protein ligase in locus 1-1. A rice U-box E3 ubiquitin ligase (*OsPUB67*) was significantly induced by drought, and its overexpression enhances the reactive oxygen species scavenging ability and stomatal closure, which improves drought tolerance (Qin et al., 2020). For antioxidant activities, we found a UDP-glucosyl transferase (UGT) in locus 4-2. Overexpressing two *Arabidopsis* UGTs, *UGT79B2* and *UGT79B3*, increases drought tolerance thanks to increased anthocyanin accumulation and enhanced antioxidant activity in coping with drought (Li et al., 2017), and similar results have also been reported in rice (Dong et al., 2020).

The last candidate gene to be described is *jasmonic acid-amino synthetase1* (*JAR1*) in locus 5-2. Jasmonic acid (JA) is of central importance in drought stress responses (Wasternack, 2014). *JAR1* is involved in conjugating JA to Ile, the bioactive form of JA (Staswick and Tiryaki, 2004). *JAR1* plays a major role in JA signaling (Kazan and Manners, 2008), and its expression is upregulated in the early stages of drought and decreased upon persistent drought (Chen et al., 2019). Overexpressing *JAR1* reduces water loss during drought, while its mutation lowers JA–Ile content and causes hypersensitivity to drought (Mahmud et al., 2022).

In conclusion, we evaluated a sorghum mini core panel for tolerance to drought simulated by PEG. We confirmed results from previous studies that sorghum plants produced more roots than longer roots at 10% PEG, but at 20% PEG, they produced longer roots than more roots, and PEG reduced shoot growth in all accessions in both years. GWAS identified 22 loci, 19 of which were mapped to the STC traits, and 17 of the 19 were mapped to the STC of shoot weight. Eleven of the 22 loci were colocated with 15 QTLs that had been previously mapped to green leaf area, the total number of green leaves, or chlorophyll content. Of the 19 candidate genes from the 12 loci mapped, five showed either preferential or specific expression in the roots according to GeneAtlas v2. One of the candidate genes from locus 6-1, colocated with a previously mapped chlorophyll fluorescence QTL, was found to increase chlorophyll fluorescence in another study. Sorghum leaf chlorophyll content is closely associated with drought tolerance. IS 30533 was the most tolerant accession, and IS 32439 was the most sensitive accession. The results from this study will facilitate sorghum marker-assisted breeding for drought tolerance.

## Data Availability

The datasets presented in this study can be found in online repositories. The names of the repository/repositories and accession number(s) can be found in the article/**Supplementary Material**.
