# Supplementary figures and images for: Association mapping and candidate gene identification for drought tolerance in sorghum
Source: Front Plant Sci. 2025 Jul 25;16:1629615. doi: 10.3389/fpls.2025.1629615 (PMC12331711; doi:10.3389/fpls.2025.1629615)

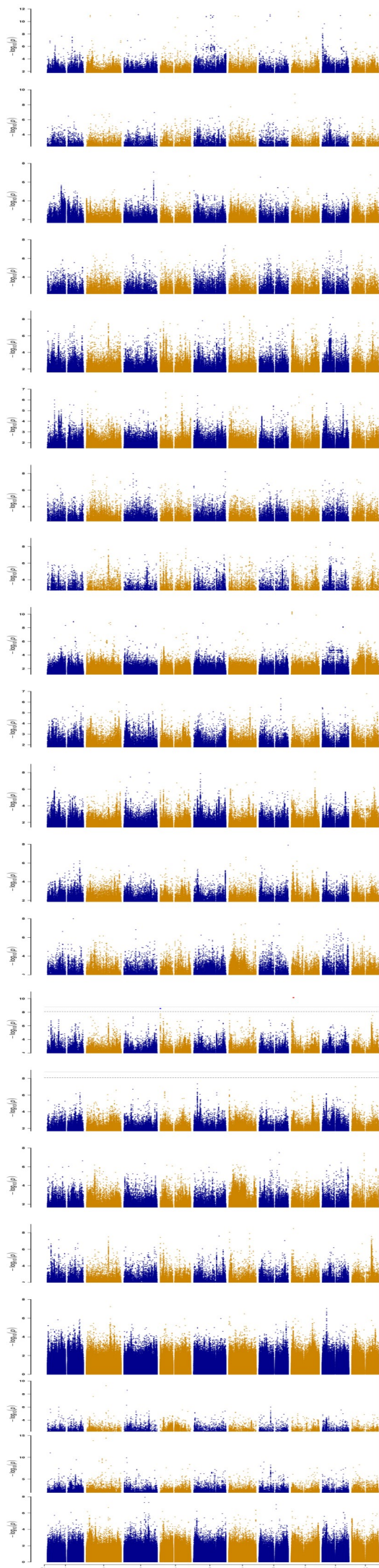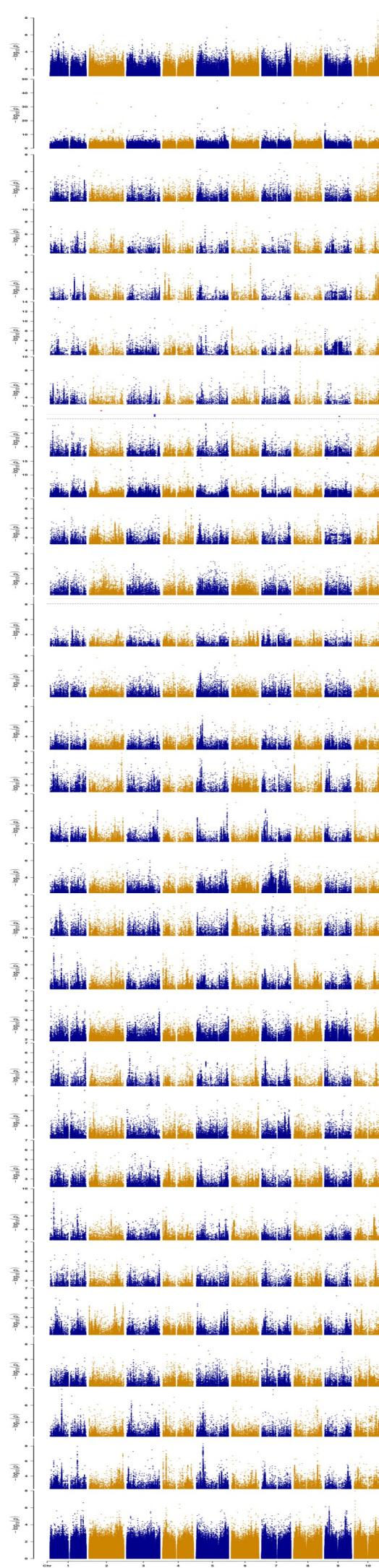

Supplement: Supplementary file 1 [file Image1.pdf]
